# A molecular electron density theory study of the participation of tetrazines in aza-Diels–Alder reactions†

**DOI:** 10.1039/d0ra01548b

**Published:** 2020-04-21

**Authors:** Luis R. Domingo, Mar Ríos-Gutiérrez, Patricia Pérez

**Affiliations:** Department of Organic Chemistry, University of Valencia Dr Moliner 50, 46100 Burjassot Valencia Spain domingo@utopia.uv.es; Department of Chemistry and Chemical Biology, McMaster University 1280 Main Street West Hamilton Ontario L8S 4L8 Canada; Universidad Andres Bello, Facultad de Ciencias Exactas, Departamento de Ciencias Químicas, Computational and Theoretical Chemistry Group Av. República 498 8370146 Santiago Chile p.perez@unab.cl

## Abstract

The reactions of eight tetrazines of increased electrophilic character with nucleophilic tetramethyl ethylene (TME) and with electrophilic tetracyanoethylene (TCE) have been studied using Molecular Electron Density Theory. These reactions are domino processes comprising an aza-Diels–Alder (ADA) reaction followed by an extrusion of molecular nitrogen, yielding a dihydropyridazine. Analysis of the conceptual DFT (CDFT) indices showed an increase of the electrophilicity and a decrease of the nucleophilicity of tetrazines with an increase of the electron-withdrawing character of the substituent. A very good correlation between the global electron density transfer at the transition structures and the activation enthalpies for the ADA reactions involving TME was found. However, tetrazines have no tendency to react with electrophilic ethylenes such as TCE. Bonding Evolution Theory (BET) analysis of the ADA reaction of dinitro tetrazine with TME showed that the activation energy is mainly associated with the continuous depopulation of the C–C and C–N double bonds.

## Abstract 2

The electron-withdrawing substituents on the tetrazine favour aza-Diels–Alder reactions towards nucleophilic ethylenes, but they do not react with electrophilic ethylenes.

## Graphical abstract

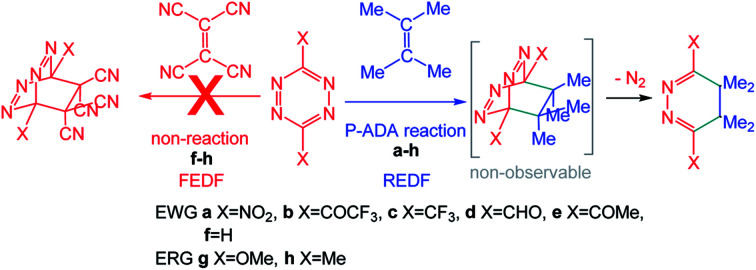

## 1. Introduction

**Scheme 1:**
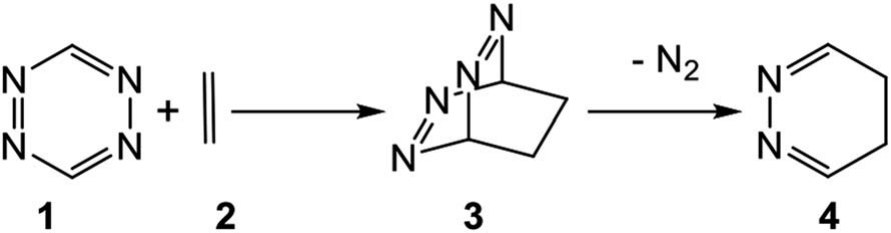
Obtaining pyridazine 4 through an ADA reaction of tetrazine 1 with ethylene 2 and subsequent loss of molecular nitrogen at bicyclic adduct 3.

1,2,4,5-Tetrazine 1 has proven to be a useful reagent that participates in aza-Diels–Alder (ADA) reactions with a wide range of ethylene and acetylene derivatives, providing rapid access to a range of highly substituted pyridazines.^1–8^ The expected bicyclic compounds such as 3 have never been observed, with pyridazine 4 being the product obtained by loss of molecular nitrogen (see Scheme 1).

In general, 3,6-disubstituted tetrazines are largely employed due to their synthetic accessibility. Trends in reactivity of tetrazines have been explored in some detail by Boger and co-workers,^9^ introducing several symmetrical^10^ and asymmetrical^11^ 3,6-disubstituted tetrazines.

**Scheme 2:**
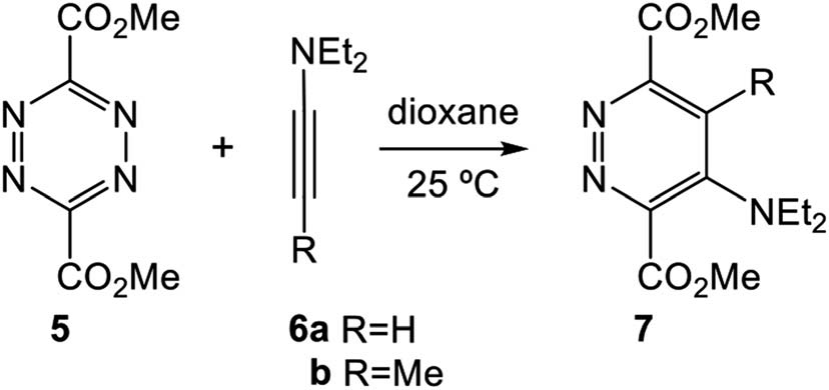
ADA reactions of tetrazine 5 with alkynes 6 yielding pyridazines 7, studied by Birney *et al.*

The ADA reactions of some simple tetrazines with acetylene derivatives to yield pyridazines have been studied theoretically.^12–14^ In 2006, Birney *et al.* reported an experimental and DFT theoretical study about the sequential transition state structures (TSs) involved in the reactions of symmetric methyl dicarboxylate tetrazine 5 with the alkynes 6 yielding pyridazines 7 (see Scheme 2).^14^ Kinetic measurements of the reaction of tetrazine 5 with 6b gave an activation Gibbs free energy of 11.5 kcal mol^−1^. Calculations at the B3LYP/6-31G(d,p) level for the ADA reaction of tetrazine 5 with 6b showed that it takes place through a one-step mechanism involving a very asynchronous TS (1.892 and 2.944 Å); the computed barrier of the ADA reaction was 7.6 kcal mol^−1^.

**Scheme 3:**
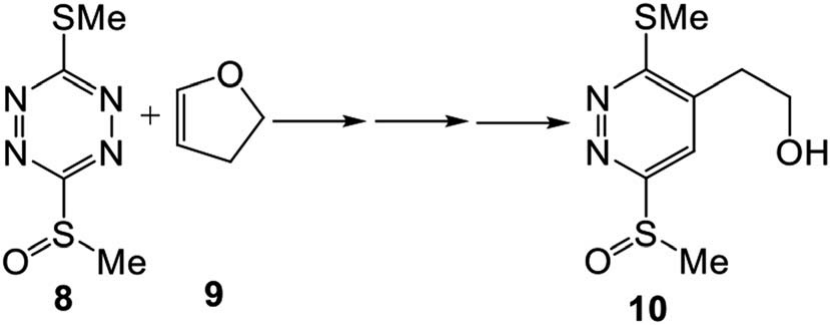
Reaction between electrophilic disubstituted tetrazine 8 and nucleophilic cyclic vinyl ether 9.

Unlike the ADA reactions of tetrazines with acetylenes, ADA reactions with ethylenes have not been theoretically treated in detail. In 2008, the unexpected regioselective ADA reactions of non-symmetric tetrazines with nucleophilic ethylenes, experimentally studied by Boger,^15^ were studied at the B3LYP/6-31G(d) computational level (see Scheme 3).^16^ That work emphasized that the high electrophilic character of disubstituted tetrazine 8 together with the high nucleophilic character of cyclic vinyl ether 9 favour this ADA reaction through a polar mechanism.^17^ The unexpected regioselectivity of these ADA reactions was explained by a polar mechanism. Although the nucleophilic attack of vinyl ether 9 over the *para* position relative to the methylsulfinyl substituent of tetrazine 8 favours the global electron density transfer (GEDT), it is energetically more unfavourable because it diminishes the electron density at the electrophilic tetrazine core.^16^

In 2014, Houk *et al.* studied the ADA reactions of seven tetrazines with unstrained and strained alkenes and alkynes, using M06-2X/6-31G(d) calculations.^18^ After a Distortion/Interaction Model (DIM)^19,20^ analysis, they suggested that “the reactivity of the substituted tetrazines correlates well with the electron-withdrawing (EW) character of the substituents. EW groups lower the LUMO+1 of tetrazines, resulting in stronger interactions with the HOMO of dienophiles. Moreover, EW substituents destabilize tetrazines, which leads to smaller distortion energies in the Diels–Alder transition states”, and “alkenes have HOMO energies higher than those of alkynes and therefore stronger interaction energies in inverse electron-demand Diels–Alder reactions with tetrazines.” More recently, Houk *et al.* also applied the DIM to the Diels–Alder reactivities of benzenes and ten azabenzenes, including pyridines, diazines, triazines and tetrazines, ascribing their reactivities toward ethylene to the distortion energies.^21^

In this context, it is worth highlighting that MOs are mathematical constructs, not physically observable, proposed in 1932 for the construction of the wave function only.^22^ In addition, within the DFT framework,^23^ Kohn–Sham orbitals do not define any wave function, although they are used to calculate the one-electron density distribution function.^24^ In contrast, the electron density distribution in a molecule or crystal can be determined by electron diffraction and X-ray crystallography,^25^ and it can also, and often quite more readily, be obtained from *ab initio* or DFT calculations.^26^

On the other hand, Houk's DIM,^19,20^ based on the Energy Decomposition Analysis (EDA) scheme proposed by Morokuma in 1981,^27,28^ has no quantum physical meaning within DFT, since in DFT the energy of a system is a functional of the electron density and the external potential,^23^ and consequently, the energy of the constrained separated geometries conforming the TS has no relationship with the energy of the actual TS as each of them loses the external potential created by the other fragment.^29,30^

In 2016, Domingo proposed the Molecular Electron Density Theory^31^ (MEDT) for the study of the reactivity in Organic Chemistry, in which changes in the electron density, but not MO interactions, as the Frontier Molecular Orbital (FMO) theory proposes,^32^ are responsible for the feasibility of an organic reaction. A thorough study of experimental Diels–Alder reactions allowed establishing a linear relationship between activation energies and the polar character of the reactions, measured through the GEDT^33^ taking place at the TSs, making it possible to establish the polar Diels–Alder (P-DA) mechanism, in which the activation energies depend mainly on the nucleophilic/electrophilic behaviours of the reagents.^17^ The Conceptual DFT (CDFT) indices, such as the electrophilicity *ω* and nucleophilicity *N* indices, have become powerful quantum chemical tools to predict the polar character of DA reactions, and thus, their feasibility; *i.e.* the more nucleophilic the diene and more electrophilic the ethylene, or *vice versa*, the more polar and faster the DA reaction.^17^

**Chart 1:**
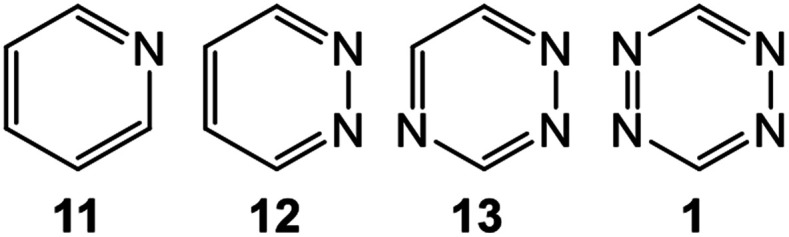
Series of AACs 1, 11–13 participating in ADA reactions.

Very recently, the enhanced reactivity of a series of aza aromatic compounds (AACs) 1, 11–13 participating in the ADA reactions with ethylene was studied within MEDT.^34^ The replacement of C–H unities by N: ones increases the reactivity of these species as the ring electron density (RED) decreases. This behaviour triggers two cooperative effects in the reduction of the activation energies of the ADA reactions: (i) the loss of the aromatic character of these AACs, which thermodynamically destabilizes the reagents; and (ii) the increase of the electrophilic character of the AACs, which increases the polar character of the ADA reactions (Chart 1).

ADA reactions of tetrazines have been classified as “inverse electron-demand (IED) Diels–Alder reactions” within the FMO theory.^35^ In 1973, Houk renamed Sustmann's classification,^35^ naming it (HOMO) HO- and (LUMO) LU-controlled cycloaddition reactions,^36^ emphasising the relevance of the MO interactions in cycloaddition reactions. However, neither classification based on the FMO theory has any chemical significance. Thus, for instance, the unfavourable DA reaction between butadiene and ethylene was classified as direct electron-demand (DED) or HO-controlled, despite this non-polar DA reaction having a negligible GEDT at the corresponding TS.^37^ In addition, many authors have emphasised that these classifications are sometimes confusing, leading to interpretations in contrast with the experimental observations.^38–41^

At the TSs of polar cycloaddition reactions, the electron density always fluxes from the nucleophilic species to the electrophilic ones. The CDFT^42,43^ provides a series of global reactivity indices such as the electronic chemical potential *μ*,^44,45^ the electrophilicity *ω*^46^ and nucleophilicity *N*^47^ indices, which unequivocally permit the establishment of the polar character of cycloadditions and the direction of the electron density flux; in a polar reaction the electron density always fluxes from the species with higher electronic chemical potential *μ* towards the species with lower electronic chemical potential *μ*.^48,49^ In the cases in which the two reagents present similar electronic chemical potential *μ*, the CDFT predicts the reaction to be non-polar.

Due to synthetic significance of the characterisation of the direction of the electron density flux in polar cycloaddition reactions, since it allows identifying the nucleophilic and electrophilic species participating in the reaction, we propose herein to refer to reactions of forward electron density flux (FEDF) to those in which the electron density fluxes from a nucleophilic diene or a three-atom-component (TAC) participating in a [3 + 2] cycloaddition reactions towards an electrophilic ethylene, and reactions of reverse electron density flux (REDF) when the electron density fluxes from a nucleophilic ethylene towards an electrophilic diene or TAC. Note that this classification is completely arbitrary when choosing the diene or the TAC as the electron donor in an FEDF reaction.

**Scheme 4:**
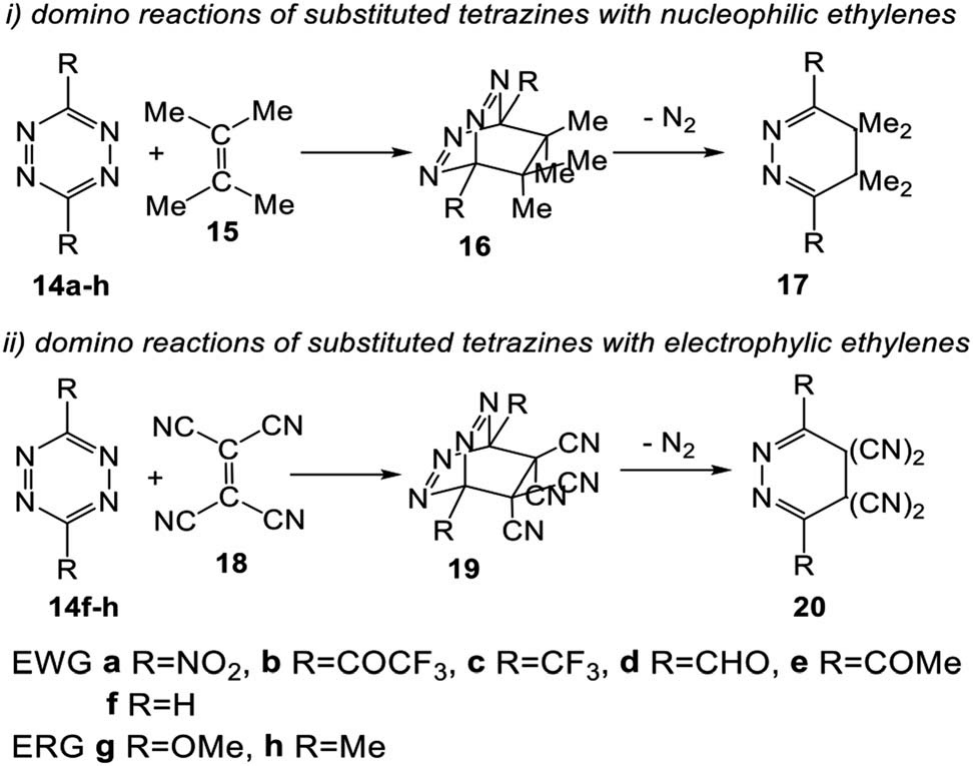
Domino reactions of tetrazines 14 with TME 15 and TCE 18 herein studied.

Herein, an MEDT study of the domino reactions of a series of symmetrically disubstituted tetrazines 14a–h of increased electrophilic character toward strong nucleophilic tetramethyl ethylene (TME) 15 is carried out in order to rationalize the increased reactivity of this series of tetrazines participating in ADA reactions towards nucleophilic ethylenes (see Scheme 4). In addition, the reaction of tetrazines 14f–h, the most nucleophilic tetrazines of this series, with tetracyanoethylene (TCE) 18, one of the most electrophilic ethylenes, is also studied in order to shed light on the response of tetrazines towards electrophilic ethylenes (see Scheme 4).

## 2. Computational details

DFT calculations were performed using the MPWB1K functional^50^ together with the 6-311G(d,p) basis set.^26^ Full optimisations at this level of theory were carried out using the Berny analytical gradient optimisation method.^51,52^ The stationary points were characterised by frequency computations in order to verify that TSs have one and only one imaginary frequency. The IRC paths,^53^ in amu^1/2^ bohr units, were traced in order to check the energy profiles connecting each TS to the two associated minima using the second order González–Schlegel integration method.^54,55^ Solvent effects of dichloromethane (DCM) were taken into account by full optimisation of the gas phase geometries through the polarisable continuum model^56,57^ (PCM) developed within the self-consistent reaction field^58–60^ (SCRF). Values of enthalpies, entropies and Gibbs free energies in DCM were calculated with standard statistical thermodynamics at 25 °C and 1 atm.^26^

CDFT reactivity indices^42,43^ were computed as stated in ref. 43. GEDT^33^ was computed as the sum of the natural atomic charges (*q*), obtained by a Natural Population Analysis^61,62^ (NPA), of the atoms belonging to each framework (f) at the TSs; *i.e.*
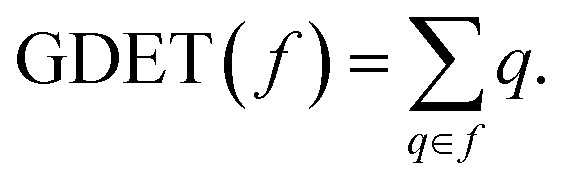
 Positive values mean a flux from the considered framework to the other one. The Gaussian 16 suite of programs^63^ was employed in all computations.

Electron Localisation Function^64^ (ELF) studies were performed with the TopMod package,^65^ considering the standard cubical grid of step size of 0.1 bohr. The bonding changes along the corresponding reactions were analysed, according to the Bonding Evolution Theory^66^ (BET), by performing the topological analysis of the ELF for 690 nuclear configurations along the IRC path.

The molecular geometries and ELF basin attractor positions were visualised using the GaussView program,^67^ while the ELF localisation domains were represented by using the Paraview software at an isovalue of 0.75 a.u.^68,69^

## 3. Results ad discussion

The present MEDT study has been divided in five sections: in Section 3.1, an analysis of the CDFT reactivity indices at the ground state (GS) of the reagents is presented. In Section 3.2, the domino reactions of the series of tetrazine derivatives with nucleophilic TME 15 are explored. In Section 3.3, the study of the domino reactions of tetrazines 14f–h with electrophilic TCE 18 is performed. In Section 3.4, a BET study of the ADA reaction between dinitro tetrazine 14a and TME 15 is carried out in order to characterize the computed high reactivity. Finally, in Section 3.5, an ELF analysis of the TSs involved in the ADA reactions of disubstituted tetrazines 14a–h and TME 15 is performed in order to obtain additional insight into the different reactivities of the tetrazine series.

### 3.1. Analysis of the CDFT reactivity indices at the GS of the reagents

First, in order to understand the polar nature of these ADA reactions, an analysis of the CDFT indices^42,43^ at the GS of the reagents computed at the B3LYP/6-31G(d) level was performed. The reason for the choice of this computational method is that the original reactivity scales within which the reagents of this study will be compared were established based on this method. The global CDFT indices, namely, electronic chemical potential *μ*, chemical hardness *η*, global electrophilicity *ω*, and global nucleophilicity *N*, of the reagents are gathered in Table 1. The MPWB1K/6-311G(d,p) global CDFT indices are given in Table S1 in ESI.†

**Table 1:** B3LYP/6-31G(d) electronic chemical potential *μ*, chemical hardness *η*, global electrophilicity ω, and global nucleophilicity *N*, in eV, for the studied reagents

	R	*μ*	*η*	*ω*	*N*
TCE 18		−7.04	4.17	5.95	0.00
14a	NO_2_	−6.59	3.63	5.99	0.72
14b	COCF_3_	−6.09	3.26	5.69	1.40
14c	CF_3_	−5.85	3.66	4.68	1.44
14d	CHO	−5.71	3.44	4.74	1.70
14e	COMe	−5.26	3.53	3.91	2.10
14f	H	−4.98	3.67	3.38	2.30
14g	OMe	−4.63	3.65	2.93	2.67
14h	Me	−4.50	3.58	2.83	2.83
Ethylene 2		−3.37	7.77	0.73	1.87
TME 15		−2.46	6.94	0.43	3.20

The electronic chemical potential^44,45^*μ* of TME 15, −2.46 eV, is very high with respect to that of the substituted tetrazines 14a–h, between −4.50 (14h, R = Me) and −6.59 (14a, R = NO_2_) eV, indicating that the GEDT in these polar ADA reactions will take place from TME 15 to these tetrazine derivatives. Thus, these reactions are classified as of REDF. On the other hand, the electronic chemical potentials *μ* of tetrazines 14f–h, between −4.98 eV (14f, R = H) and −4.50 (14h, R = Me), are higher than that of TCE 18, −7.04 eV, indicating that the GEDT in these polar ADA reactions will take place from the tetrazines to TCE 18. Thus, these reactions are classified as FEDF reactions.

The electrophilicity^46^*ω* and nucleophilicity^47^*N* indices of the simplest tetrazine 14f are 3.38 and 2.30 eV, respectively, being classified as a strong electrophile and a moderate nucleophile within the electrophilicity and nucleophilicity scales.^43^ The electrophilicity *ω* index of the EW substituted tetrazines ranges from 3.91 eV (14e, R = COMe) to 5.99 eV (14a, R = NO_2_), being classified as strong electrophiles. As expected, a clear correlation between the EW character of the substituent and the increase of the electrophilicity of the tetrazine derivative is observed. On the other hand, the nucleophilicity *N* index of these species ranges from 0.27 eV (14a, R = NO_2_) to 2.10 eV (14e, R = COMe), being classified as marginal nucleophiles. Consequently, the inclusion of two EW groups in the tetrazine core considerably increases the electrophilicity of the corresponding tetrazine derivatives, suggesting that the corresponding REDF ADA reactions with nucleophilic ethylenes will take place very easily.

The electrophilicity *ω* indices of the ER substituted tetrazines are 2.93 eV (14g, R = OMe) and 2.83 eV (14h, R = Me), also being classified as strong electrophiles. However, the nucleophilicity *N* indices of these species are 2.67 eV (14g, R = OMe) and 2.83 eV (14h, R = Me), being classified as moderate nucleophiles. As expected, the inclusion of two ER groups in the tetrazine core decreases the electrophilic character of the corresponding tetrazine derivatives, although they remain strong electrophiles, and increases their nucleophilic character, enabling them to react easily towards electrophilic ethylenes.

The electrophilicity *ω* and nucleophilicity *N* indices of ethylene 2 are 0.73 and 1.87 eV, respectively, being classified as a marginal electrophile and a marginal nucleophile. The inclusion of four ER methyl substituents in ethylene 2 decreases the electrophilicity *ω* index of TME 15 to 0.43 eV and considerably increases the nucleophilicity *N* index to 3.20 eV, being classified as a strong nucleophile. Thus, while ethylene 2 does not participate in polar reactions, TME 15 will participate in polar reactions as a strong nucleophile. On the other hand, TCE 18, with an electrophilicity *ω* index of 5.95 eV, is one of the most electrophilic neutral organic molecules.

### 3.2. Study of the domino reactions of the series of tetrazine derivatives 14a–h with TME 15

**Scheme 5:**
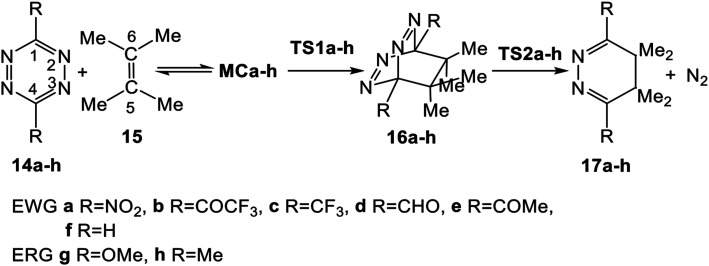
Domino reactions of the series of tetrazine derivatives 14a–h with TME 15.

The reactions of the series of tetrazines 14a–h with TME 15 yielding dihydropyridazine 17a–h and molecular nitrogen were studied in DCM. These reactions are domino processes that comprise three consecutive steps (see Scheme 5): (i) formation of molecular complexes (MCs) MCa–h; (ii) an FEDF ADA reaction from these MCs yielding the bicyclic compounds 16a–h; and, finally, (iii) an extrusion of molecular nitrogen in these bicyclic compounds affording dihydropyridazine 17a–h. Relative enthalpies and Gibbs free energies in DCM are given in Table 2.

**Table 2:** MPWB1K/6-311G(d,p) relative enthalpies (Δ*H*, in kcal mol^−1^) and Gibbs free energies (Δ*G*, in kcal mol^−1^), computed at 25 °C and 1 atm in DCM, for the stationary points involved in the domino reactions of the series of tetrazine derivatives 14a–h with TME 15

	R=	14a NO_2_	14b COCF_3_	14c CF_3_	14d CHO	14e COMe	14f H	14g OMe	14h Me
Δ*H*	MC	−4.4	−4.0	−3.4	−3.9	−4.1	−2.3	−2.4	−2.5
	TS1	1.1	6.8	10.9	9.8	11.6	13.9	21.4	20.7
	16	−33.1	−22.0	−25.2	−18.4	−17.0	−19.9	−12.5	−11.7
	TS2	−17.6	−9.6	−10.9	−7.7	−5.1	−5.3	−4.2	1.8
	17	−74.4	−67.5	−66.9	−59.6	−60.8	−57.3	−64.6	−53.0
Δ*G*	MC	8.2	7.6	8.7	8.3	6.9	7.4	9.2	9.9
	TS1	17.8	23.2	27.9	25.6	27.7	28.3	36.8	36.4
	16	−14.9	−3.0	−7.6	−0.8	0.8	−4.0	4.7	5.4
	TS2	−0.3	7.9	6.7	8.6	11.1	10.3	12.3	18.0
	17	−70.2	−63.2	−62.3	−56.3	−57.4	−53.9	−60.7	−48.6

These domino reactions begin with the formation of an MC in which TME 15 is oriented in a parallel rearrangement relative to the aromatic ring of tetrazine derivatives 14a–h (see the structures of MCa and MCh in Fig. S1 in ESI†). The subsequent approach of the two reagents in these MCs allows reaching the TSs of the ADA reactions, TS1a–h, which in an elementary step yield the corresponding bicyclic compounds 16a–h. Finally, the second reaction of these domino processes is the loss of molecular nitrogen *via* a retro ADA reaction in these bicyclic compounds, yielding the final dihydropyridazines 17a–h plus molecular nitrogen.

Formation of the MCs of the ADA reactions are exothermic between 2.3 (MCf, R = H) and 4.4 (MCa, R = NO_2_) kcal mol^−1^. From these species, the activation enthalpies associated with the TSs range from 5.5 (TS1a, R = NO_2_) to 23.8 (TS1g, R = OMe) kcal mol^−1^. From the separated reagents, formation of the bicyclic compounds 16a–h is exothermic between 11.7 (16h, R = Me) and 33.1 (16a, R = NO_2_) kcal mol^−1^. From these bicyclic compounds, the extrusion of the molecular nitrogen yielding the final dihydropyridazine 17a–h has an activation enthalpy ranging from 8.3 (TS2g, R = OMe) to 15.5 (TS2a, R = NO_2_) kcal mol^−1^. The overall domino processes are exothermic between 53.0 (17f, R = H) and 74.4 (17a, R = NO_2_) kcal mol^−1^.

Some appealing conclusions can be drawn from these energy values: (i) formation of these MCs presents a similar exothermic character along this series, indicating that their formation is not significantly affected by the electronic nature of the substituent present in the tetrazine core. Note that their formation is found in the narrow range of −2.3 and −4.4 kcal mol^−1^; (ii) while the EW substitution on the tetrazine core decreases the activation enthalpy associated with the ADA reactions with respect to that of simplest tetrazine 1, 16.2 kcal mol^−1^, the ER substitution increases it; (iii) a good correlation between the increase of the electrophilic character of these tetrazine derivatives and the decrease of the activation energies can be established; (iv) in the same way, an increase of the exothermic character of these ADA reactions is also observed with the increase of the EW character of the substituents; and finally, (v) the activation enthalpies associated with the extrusion of molecular nitrogen are found to be less dependent on the substitution of the tetrazine core than the ADA reactions. Thus, while the activation enthalpies associated with these ADA reactions are around 18.8 kcal mol^−1^, those associated with the extrusion of molecular nitrogen are around 7.2 kcal mol^−1^.

**Fig. 1:**
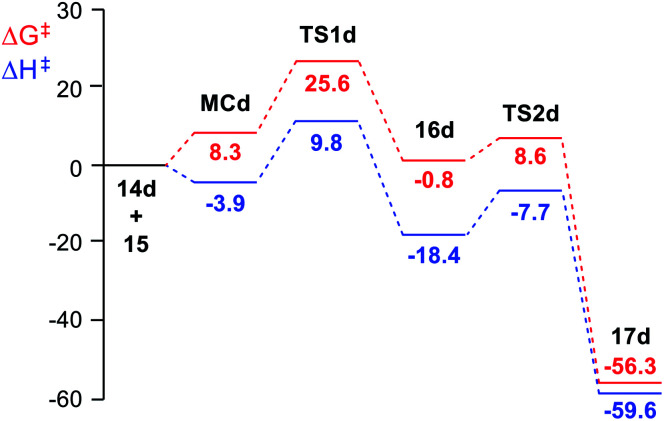
MPWB1K/6-311G(d,p) enthalpy, in blue, Δ*H* in kcal mol^−1^, and Gibbs free energy, in red, Δ*G* in kcal mol^−1^, profiles, in DCM at 25 °C, for the domino reaction of tetrazine derivative 14d (R = CHO) with TME 15 yielding dihydropyridazine 17d.

Inclusion of the term *T*Δ*S* to the enthalpies increases the relative Gibbs free energies of the stationary points by between 10 and 19 kcal mol^−1^, given the bimolecular nature of the reaction (see Fig. 1). In the Gibbs free energy profile, formation of the MCs is endergonic by 7.4 (MCf, R = H) and 9.9 (MCh, R = Me) kcal mol^−1^. As a consequence, the activation Gibbs free energies for the first ADA reactions are found between 17.8 (TS1a, R = NO_2_) and 36.8 (TS1g, R = OMe) kcal mol^−1^. While the formation of the bicyclic compounds 16d, 16f, and 16g is endergonic by 0.8–5.4 kcal mol^−1^, formation of the other bicyclic compounds is exergonic by 0.8–14.9 kcal mol^−1^. The activation Gibbs free energy for the subsequent extrusion of molecular nitrogen *via*TS2 is found to be between 7.6 (TS2g, R = OMe) and 14.6 (TS2a, R = NO_2_) kcal mol^−1^. Finally, formation of the dihydropyridazines is strongly exergonic, between 48.6 (16h, R = Me) and 70.2 (16a, R = NO_2_) kcal mol^−1^; these domino processes being irreversible. In the eight domino reactions, the activation Gibbs free energy associated with the ADA reaction is higher than that associated to the extrusion of molecular nitrogen; consequently, the ADA reaction is the rate determining step (RDS) of these domino processes.

The enthalpy and Gibbs free energy profiles of the domino reaction of tetrazine 14d (R = CHO) with TME 15 yielding dihydropyridazine 17d, as a representative reaction of electrophilic tetrazines, is given in Fig. 1. As can be seen, while formation of MCd is exothermic by 3.9 kcal mol^−1^, its formation at 25 °C is endergonic by 8.3 kcal mol^−1^ due to of the unfavourable entropy associated with the formation of this species. The activation Gibbs free energy associated with this ADA reaction, 25.6 kcal mol^−1^, is higher than that associated with the extrusion of the molecular nitrogen at the bicyclic compound 16d, 9.4 kcal mol^−1^. The ADA reaction is the RDS of this domino process, and consequently, bicyclic compound 16d is non-observable.

**Fig. 2:**
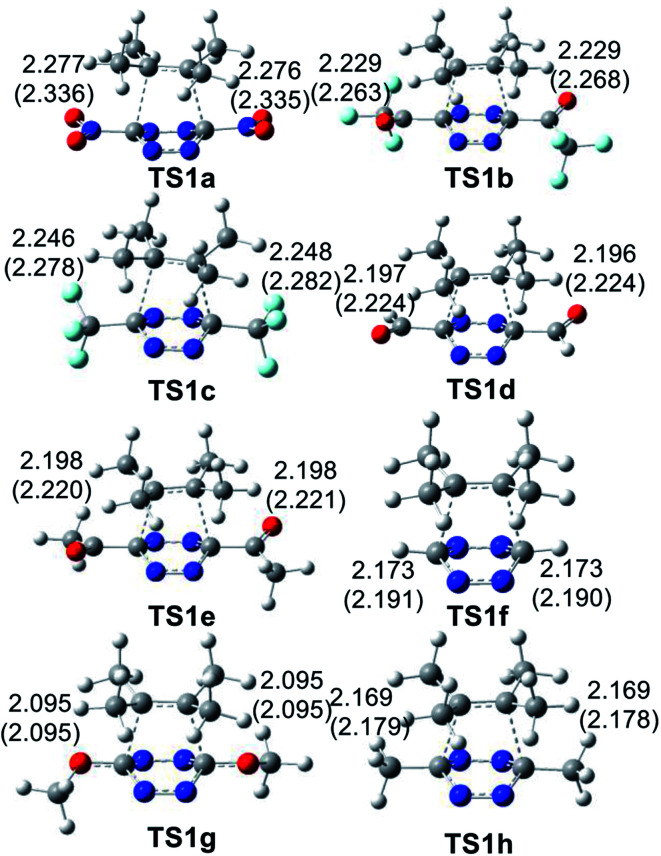
MPWB1K/6-311(d,p) geometries of the TSs involved in the ADA reactions of the series tetrazine derivatives 14a–h with TME 15. The distances are given in angstroms, Å. Values in DCM are given in parentheses.

**Fig. 3:**
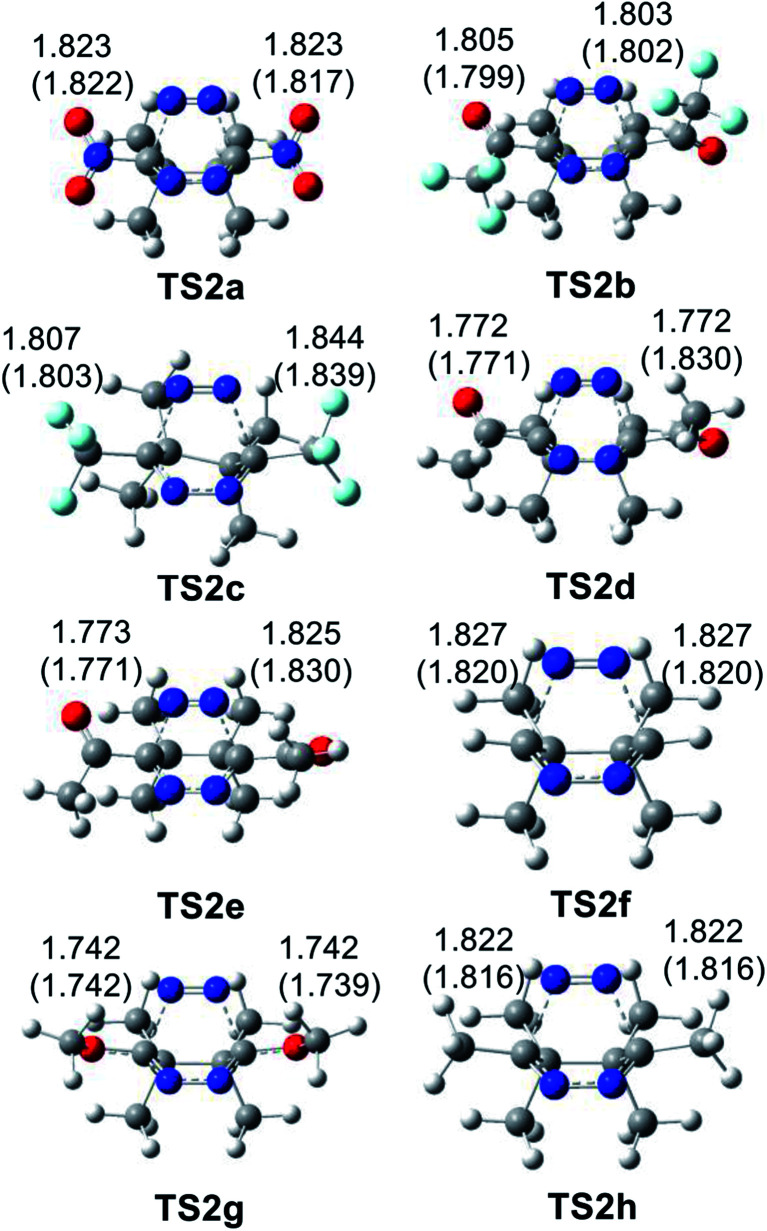
MPWB1K/6-311(d,p) geometries of the TSs involved in the retro ADA reactions of the bicyclic compounds derivatives 16a–h. Distances are given in angstroms, Å. Values in DCM are given in parentheses.

The geometries of the TSs involved in the ADA reactions of the series of tetrazines 14a–h with TME 15 are given in Fig. 2, while the geometries of the TSs involved in the extrusion of the molecular nitrogen are given in Fig. 3. The gas phase geometries of the TSs associated with the ADA reactions indicate that they are associated with synchronous C–C bond formation processes in which the distance between the interacting carbons is found in the short range of 2.10–2.28 Å. An increase of these lengths with the increase of the electrophilic character of the tetrazine derivatives and with the decrease of the activation enthalpy is observed; the simpler the ADA reactions, the earlier they will take place. Inclusion of solvent effects of DCM in the optimisation does not substantially modify the gas phase geometries. In DCM, the distances between the interacting carbons increase slightly; less than 0.1 Å.

At the eight TSs, the plain formed by the C1–C6–C5–C4 carbons is slightly distorted, between 10–15°, probably because of the presence of some non-covalent interaction taking place between the four methyl groups of TME 15 with the two substituents present in the tetrazine ring.

At the TSs associated with the extrusion of the molecular nitrogen, the lengths of the C–N breaking bonds are also found in a short range: 1.74 to 1.83 Å. These distances at the five TSs indicate that the rupture of the two C–N single bonds is slightly asynchronous.

Finally, the GEDT values at the eight TSs associated to these REDF ADA reactions in DCM, which fluxes from the TME framework to the substituted tetrazine one, are: 0.37 e at TS1a, 0.36 e at TS1b, 0.31 e at TS1c, 0.32 e at TS1d, 0.31 e at TS1e, 0.20 e at TS1f, 0.20 at TS1g and 0.21 e at TS1h. An increase of the polar character of the ADA reaction with the electrophilic character of the tetrazine derivatives is observed (see Table 1), Thus, while TS1e–g have a polar character, TS1a–d have a high polar character. The most polar TS1a corresponds to the most favourable ADA reaction.

**Fig. 4:**
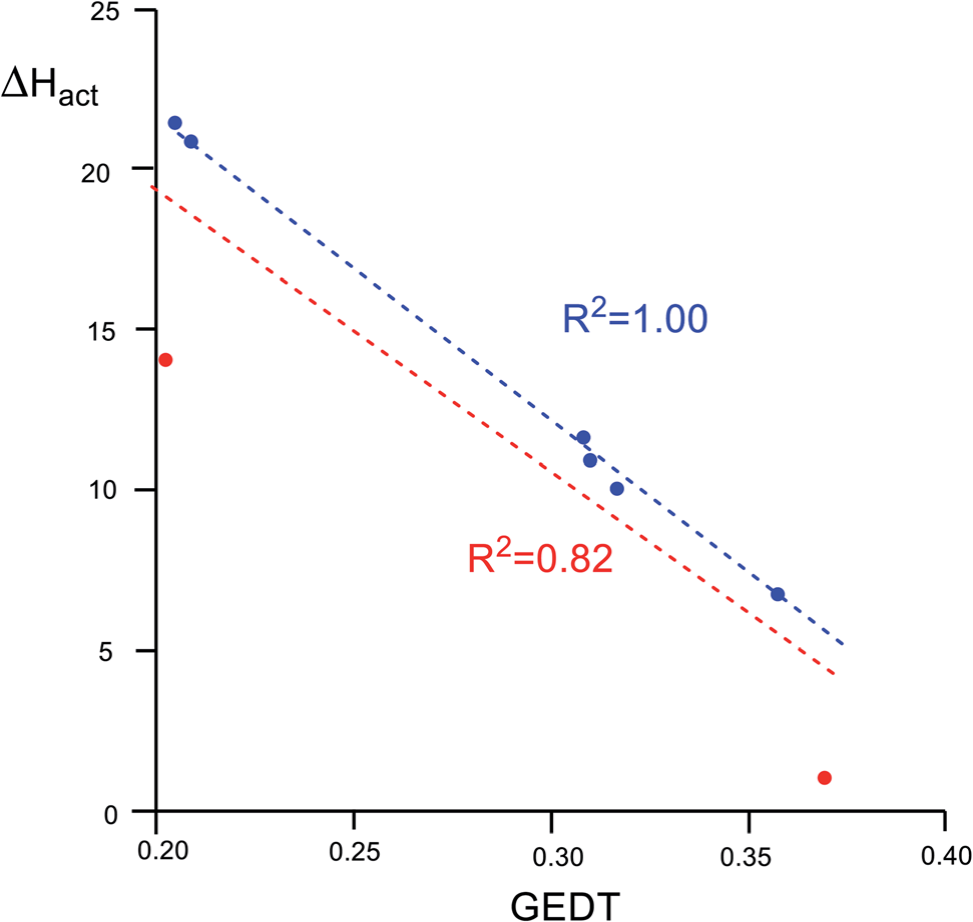
Plot of the activation enthalpies, Δ*H*_act_ in kcal mol^−1^, of the ADA reactions of disubstituted tetrazines 14a–h with TME 15*vs.* GEDT, in average number of electrons, e, computed at the corresponding TSs.

An adequate linear correlation between the activation enthalpies associated with the ADA reactions of disubstituted tetrazines 14a–h with TME 15, and the GEDT computed at the corresponding TSs is found, *R*^2^ = 0.82 (see linear correlation in red in Fig. 4). As commented on in the discussion of the geometries, a non-covalent interaction (NCI) between the TME framework and the substituents present in the tetrazine framework takes place at the TSs. Thus, when TS1f (R = H), corresponding to the unsubstituted tetrazine, and TS1a (R = NO_2_) are removed from this series, a full linear correlation with an *R*^2^ = 1.00 is found (see linear correlation in blue in Fig. 4). Consequently, the polar character of these ADA reactions, which increases with the electrophilic character of the tetrazines, appears to be a relevant electronic factor responsible for the decrease of the activation enthalpies of these ADA reactions.

### 3.3. Study of the domino reactions of the tetrazines 14f–h with electrophilic TCE 18

The domino reactions given in Scheme 5 are found in a series of disubstituted tetrazines of increased electrophilic character with a strong nucleophilic ethylene, TME 15. The corresponding polar ADA reactions are thus classified as REDF reactions. Note that the electronic chemical potentials *μ* of the tetrazines, below −4.75 eV, are below that of TME 15, *μ* = −2.64 eV. The following question can be proposed: is the participation of tetrazines in FEDF ADA reactions possible? In other word, are the P-DA reactions of tetrazines with strong electrophilic ethylenes feasible?

**Scheme 6:**
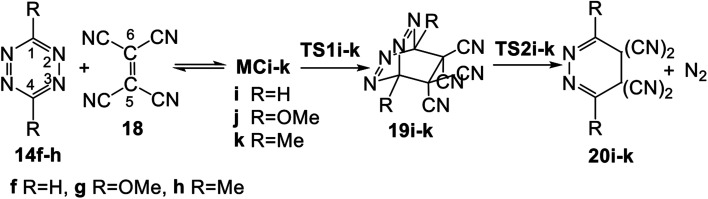
Domino reactions of tetrazines 14f–h with TCE 18.

In order to answer this question, the domino reactions between tetrazines 14f–h, the most nucleophilic tetrazines given in Table 1, and TCE 18, one of the most electrophilic ethylenes, were studied (see Scheme 6). Note that the electronic chemical potentials *μ* of tetrazine 14f–h, between −5.17 and −4.75 eV, are higher than that of TCE 18, −7.35 eV (see Table 1). Relative enthalpies and Gibbs free energies in DCM are given in Table 3.

**Table 3:** MPWB1K/6-311G(d,p) relative enthalpies (Δ*H*, in kcal mol^−1^) and Gibbs free energies (Δ*G*, in kcal mol^−1^), computed at 25 °C and 1 atm in DCM, for the stationary points involved in the domino reactions of the series of tetrazine derivatives 14f–h with TCE 18

	R=	14f H	14g OCH_3_	14h CH_3_
Δ*H*	MC	−4.2	−5.0	−5.3
	TS1	33.4	34.2	30.0
	19	2.9	12.8	2.7
	TS2	14.5	18.1	14.6
	20	−39.6	−45.6	−41.7
Δ*G*	MC	5.4	4.8	5.7
	TS1	48.1	49.5	45.1
	19	18.3	27.7	20.4
	TS2	29.5	33.2	29.5
	20	−37.1	−42.8	−38.4

Formation of the MCs MCi–k is exothermic between 4.2 (MCi, R = H) and 5.3 (MCk, R = Me) kcal mol^−1^. From these MCs, the activation enthalpies associated with the first FEDF ADA reaction are found between 39.2 (TS1j, R = OMe) and 35.3 (TS1k, R = Me) kcal mol^−1^; these ADA reactions are endothermic between 12.8 (19j) and 2.7 (19k) kcal mol^−1^. From bicyclic compounds 19i–k, the activation enthalpies associated with the extrusion of molecular nitrogen are found between 5.3 (TS2j, R = OMe) and 11.9 (TS2k, R = Me) kcal mol^−1^. The overall domino reactions are exothermic between 39.6 (20i) and 45.6 (20j) kcal mol^−1^. The inclusion of the TΔS term to the enthalpies increases the relative Gibbs free energies of MCs, TSs and bicyclic compounds between 10 and 18 kcal mol^−1^, given the bimolecular nature of the formation of MCi–k.

Some appealing conclusions can be drawn from the energies given in Table 3: (i) the activation enthalpies associated to these ADA reactions are higher than 35 kcal mol^−1^. These very high values, which are even higher than the activation energy of the non-polar Diels–Alder reaction between butadiene and ethylene, Δ*E*_act_ = 27.5 and Δ*E*_reac_ = −38.4 kcal mol^−1^, which does not takes place easily in the laboratory,^37^ prevent the corresponding FEDF ADA reaction to take place; (ii) the increase of the relative Gibbs free energies associated to the stationary points with respect to relative enthalpies, is similar to that observed in the series of domino reactions given in Scheme 5 given the bimolecular nature of the reactions. Consequently, the ADA reactions between tetrazines 14f–h and TCE 18 are the RDS of these domino processes, presenting activation Gibbs free energies above 45.1 kcal mol^−1^; (iii) formation of the bicyclic compounds 19f–h is strongly endergonic by more than 18.3 kcal mol^−1^. Note that for the series of ADA reactions given in Scheme 5, the most unfavourable of them is endergonic by 5.4 kcal mol^−1^ (14f, R = H); (iv) thus, the ADA reactions between tetrazines 14f–h and TCE 18 are both kinetically and thermodynamically very unfavourable; (v) the activation enthalpy associated with the extrusion of molecular nitrogen from bicyclic compound 19i–k, between 5–12 kcal mol^−1^, is similar to those found in the series of domino reactions given in Scheme 5; and finally, (vi) it can be concluded that the FEDF ADA reactions of tetrazines with electrophilic ethylenes are not experimentally feasible.

**Fig. 5:**
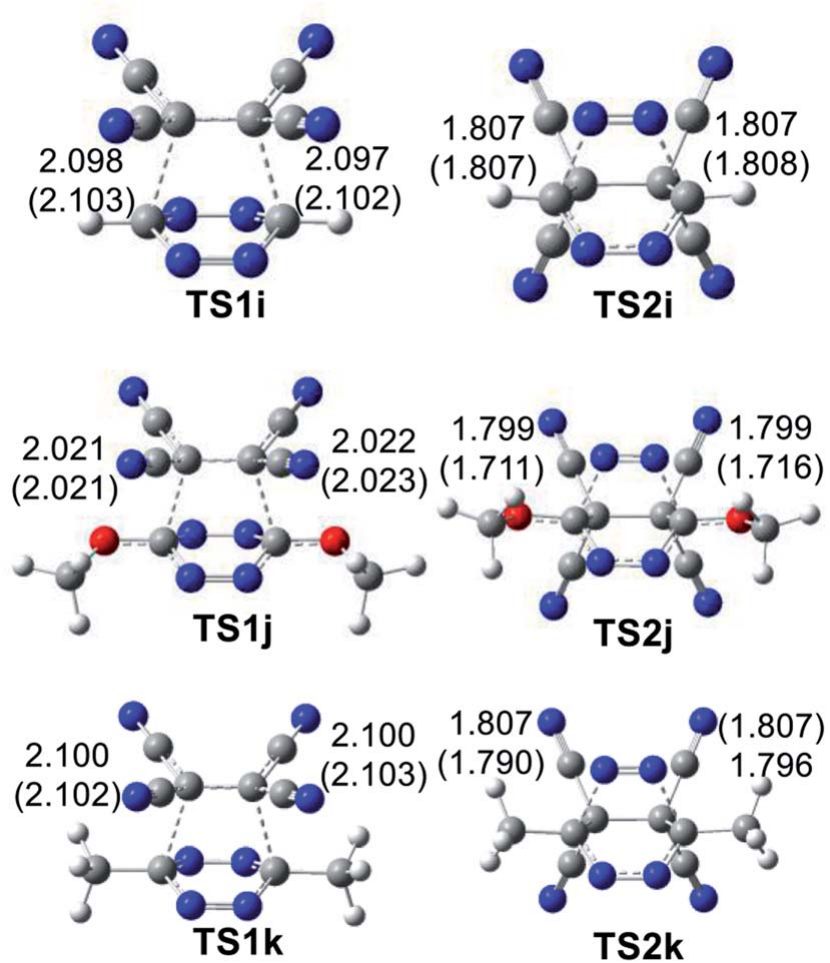
MPWB1K/6-311(d,p) geometries of the TSs involved in the domino reactions between substituted tetrazine 14f–h and TCE 18. Distances are given in angstroms, Å. Values in DCM are given in parentheses.

The geometries of the TSs involved in the domino reaction between tetrazine 14f–h and TCE 18 are given in Fig. 5. At the gas phase TSs associated to the ADA reactions, the distances between the two pairs of interacting carbons, between 2.02 and 2.10 Å, indicate that they correspond to synchronous single bond formation processes. These lengths are close to those found in the most unfavourable TS1g and TS1h. At the TSs associated with the extrusion of molecular nitrogen, the distances between the carbon and nitrogen centers are found at *ca.* 1.80 Å. These values are similar to those found in the domino reactions given in Scheme 5. Inclusion of the solvent effects of DCM does not modify the gas phase geometries substantially. Only the distances between the carbon and nitrogen centers are shortened at TS2j by 0.08 Å with the inclusion of DCM.

Finally, the GEDT values at the three TSs associated to the ADA reactions in DCM, 0.20 e (TS1i), 0.27 e (TS1j), and 0.31 e (TS1k), account for the polar character of these ADA reactions. Note that these values are similar to those found at TS1d and TS1e. In these cases, the flux of the electron density, going from tetrazines 14f–h to TCE 18, is in complete agreement with the higher electronic chemical potential μ of these tetrazines, −5.17 eV (14f), than that of TCE 18, −7.35 eV (see Table 1). Consequently, although these FEDF ADA reactions have a polar character, as a consequence of the strong electrophilic character of TCE 18, the high activation enthalpies associated to them indicate that the tetrazine core has no tendency to provide electron density in a polar process.

### 3.4. BET study of the ADA reaction between dinitro tetrazine 14a and TME 15

**Scheme 7:**
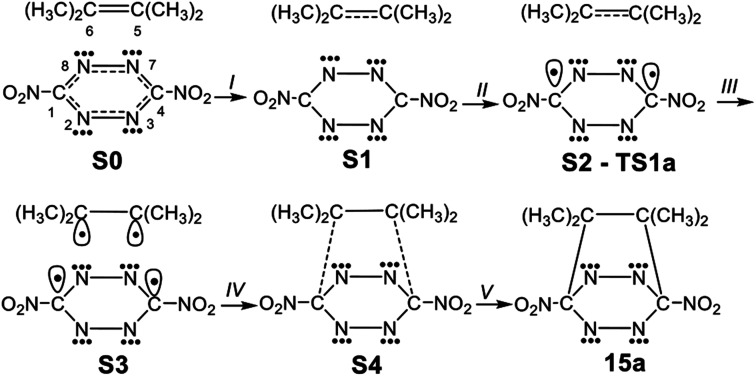
Simplified representation of the molecular mechanism of the polar DA reaction between dinitro tetrazine 14a and TME 15 by Lewis-like structures arising from the topological analysis of the ELF along the reaction path.

In order to understand the bonding changes along these REDF ADA reactions, a BET study of the polar ADA reaction of dinitro tetrazine 14a and TME 15 was carried out. The molecular mechanism represented by Lewis-like structures resulting from the ELF topology is shown in Scheme 7. Populations of the most significant valence basins of selected structures of the IRC are collected in Table 4, together with other important parameters, while the basin attractor positions are shown in Fig. S2 in ESI.†

**Table 4:** ELF valence basin populations, distances of the forming bonds, relative^a^ electronic energies, GEDT and IRC values of the IRC structures, S0–S4, defining the five phases characterising the molecular mechanism of the polar ADA reaction between dinitro tetrazine 14a and TME 15, TS1a and 16a are also included. Distances are given in angstroms, Å, GEDT values and electron populations in average number of electrons, e, relative energies in kcal mol^−1^ and IRC values in a.u.

Structures	14a	15	S0	S1	S2TS1a	S3	S4	16a
Phases			I	II	III	IV	V	
*d*(C1–C6)			2.984	2.884	2.277	2.195	2.097	1.565
*d*(C4–C5)			2.984	2.884	2.276	2.194	2.096	1.565
Δ*E*			0.0	1.1	7.8	7.4	5.1	−28.7
GEDT			0.04	0.07	0.33	0.37	0.40	0.22
IRC			−5.09	−3.20	0.00	0.46	1.24	5.92
V(C5,C6)		1.88	1.90	3.52	3.07	2.69	2.47	1.95
V′(C5,C6)		1.78	1.68					
V(C4,N7)	2.70		2.62	2.62	2.33	2.25	2.17	1.93
V(C4,N3)	2.72		2.80	2.81	2.40	2.30	2.22	1.93
V(C1,N8)	2.70		2.80	2.81	2.40	2.31	2.22	1.93
V(C1,N2)	2.72		2.62	2.61	2.34	2.25	2.17	1.93
V(N7,N8)	1.92		1.95	1.95	2.10	2.15	2.43	2.47
V(N2,N3)	1.92		1.95	1.96	2.10	2.16	2.43	2.47
V(C6)						0.18		
V(C5)						0.18		
V(C4)					0.53	0.68		
V(C1)					0.53	0.68		
V(C1,C6)							1.08	1.92
V(C4,C5)							1.08	1.92

a Relative to the first structure of the IRC, S0.

The bonding changes along this polar ADA reaction are characterised by five phases. Phase I begins at structure S0, which corresponds to that of MCa. Along this phase, a slight depopulation of the C5–C6 bonding region of the TME fragment by 0.06 e [V(C5,C6)] is observed, while the populations around the C4–N7[N3], C1–N8[N2], N7–N8 and N2–N3 bonding regions at the tetrazine moiety remain almost without changes [see Table 3].

Phase II begins at structure S1. Along this phase a depopulation of the C5–C6 bonding region of the TME fragment by *ca.* 0.45 e [V(C5,C6)] is observed.

The short Phase III starts at the structure S2, which corresponds precisely with that of TS1a. This phase begins with the creation of two C1 and C4 *pseudoradical* carbons at the tetrazine moiety, integrating 0.52 e [see V(C1) and V(C4) in Table 4 and Scheme 7]. The formation of these *pseudoradical* carbons is mainly prompted by the strong depopulation of the C4–N7[N3] and C1–N8[N2] bonding regions by *ca.* 0.30 and 0.40 e [see V(C1,N8[N2]), and V(C4,N7[N3]) in Table 4].

**Fig. 6:**
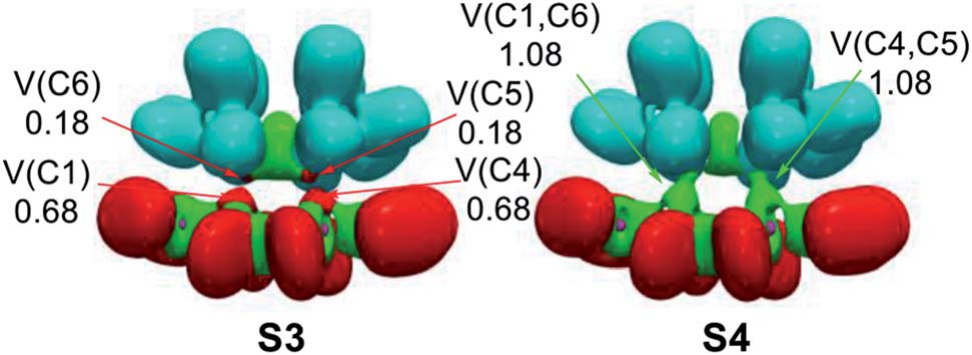
MPWB1K/6-311G(d,p) ELF localisation domains, represented at an isosurface value of ELF = 0.75; of the IRC structures S3 and S4 involved in the C–C single bond formation along the ADA reaction between dinitro tetrazine 14a and TME 15. The electron populations, in average number of electrons, is given in e.

Along Phase IV, which begins at structure S3, two new C5 and C6 *pseudoradical* carbons at the TME moiety integrating a population of 0.18 e, each one, are created [see V(C5) and V(C6) in Table 4 and in Fig. 6]. The electron density of these *pseudoradical* carbons is a consequence to the strong depopulation of the C5–C6 bonding region by *ca.* 0.38 e [V(C5,C6)]. Along this phase, it may be seen that the population associated to the C1 and C4 *pseudoradical* carbons increases to 0.68 e [see V(C1) and V(C4) in Table 4].

In the final Phase V, which begins at structure S4, and end at bicyclic compound 15a, the most relevant changes along the IRC takes place at structure S4. The two pairs of C1, C4 and C6, C5 of *pseudoradical* carbons have merged into two new C1–C6 and C4–C5 bonding regions with an initial population of 1.08 e each one. [See V(C1,C6) and V(C4,C5) in Table 4 and Fig. 6]. These electron density changes indicate that formation of the two new C–C single bonds begins at a distance of 2.10 Å by sharing the non-bonding electron densities of the two pairs of C4 and C1 towards C5 and C6 carbons. Along this phase while the population associated to the C5–C6, C4–N7[N3] and C1–N8[N2] bonding regions decreases, those associated to the N7–N8 and N2–N3 bonding regions increases by *ca.* 0.28 e.

Finally, at bicyclic compound 16a, the electron population is relaxed: the C5–C6 bonding region integrates 1.95 e, and the C4–N7[N3] and C1–N8[N2] bonding regions reach a symmetric population of 1.93 e, acquiring the expected population for single bonds, while that associated to the N8[N2]–N7[N3] bonding regions increases their populations to 2.47 e and that associated to N non-bonding electron density (not shown in the Table 4) reaches a population of 2.72 e, characterising polarized double bonds at 16a. At the end of this phase, the new C1–C6 and C4–C5 bonding regions created at S4 have reached a population of 1.92e.

From the BET analysis of the polar ADA reaction between dinitro tetrazine 14a and TME 15 some noteworthy conclusions can be drawn: (i) this polar ADA reaction takes place along five different phases. The maximum of GEDT proceeds along Phase IV (*ca.* 0.40 e). This very high GEDT is a consequence of the strong electrophilic character of dinitro tetrazine 14a; (ii) The activation energy of this reaction can mainly be associated to the continuous depopulation of the C5–C6, C4–N7[N3] and C1–N8[N2] bonding regions, which is demanded for the subsequent creation of the two C1 and C4 *pseudoradical* carbons at the tetrazine moiety at TS1a; (iii) formation of the two C1–C6 and C4–C5 single bonds takes place simultaneously at a C–C distance of 2.10 Å, by sharing the non-bonding electron densities of the two pairs of C1 and C4 towards the C6 and C5 *pseudoradical* carbons in a 78 : 22 ratio and finally, (iv) formation of the two C–C single bonds in this polar ADA is entirely synchronous.

### 3.5. ELF analysis of the TSs involved in the ADA reactions of disubstituted tetrazines 14a–h and TME 15

**Fig. 7:**
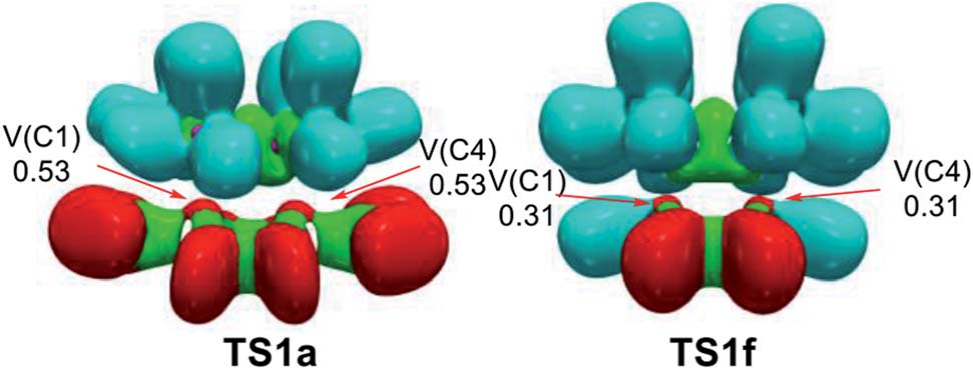
MPWB1K/6-311G(d,p) ELF localisation domains, represented at an isosurface value of ELF = 0.75; of TS1a and TS1f. The electron populations, in average number of electrons, are given in e.

Fig. S3† shows the attractor positions of the ELF valence basins of the TSs involved in the REDF ADA reactions of disubstituted tetrazines 14a–h and TME 15, while Fig. 7 shows the ELF localisation domains of TS1a and TS1f, as two representative case of this series of tetrazines.

As commented on in the previous section, ELF of the most favourable TS1a shows the presence of two monosynaptic basins, V(C1) and V(C4), at the tetrazine framework, which are associated to two C1 and C4 *pseudoradical* carbons (see Fig. 7). This picture, that is similar to the ELF of TS1b–e involving the participation of the electrophilic tetrazines 14a–e, and that of the non-substituted TS1f, indicates that they present a similar electronic structure, in complete agreement with the analysis of the geometries. Note that the C–C distances between the interacting carbons are found in the narrow range of 2.2–2.3 Å. The two C1 and C4 *pseudoradical* carbons present at TS1a have a larger population, 0.53e, than those at TS1f, 0.31 e, because of the larger GEDT taking place at the former. This behaviour provokes a stabilization of the corresponding TS, thus decreasing the electronic activation energy associated with the REDF ADA reaction.^70^

ELF of the more unfavourable TS1g and TS1h, shows the presence of four monosynaptic basins, V(C1), V(C4), V(C5) and V(C6), one pair at the tetrazine framework and other one at the ethylene, which are associated to four *pseudoradical* carbons required for the subsequent C–C single bond formation (see Fig. S3† and previous section).

This ELF analysis indicates that the more unfavourable TS1g and TS1h, involving the presence of two ER groups are slightly more advanced than TS1a–e, involving the presence of two EW groups, in clear agreement the analysis of the geometries of the TSs, which shows a shorter C–C distances at the former TSs. Consequently, the EW substitution on the tetrazine core increases the GEDT at the corresponding TS, and decreases the corresponding activation enthalpy, but does not modify the electronic structure of the TSs noticeably.

## 4. Conclusions

The reactions of eight symmetrically substituted tetrazines 14a–h of the increased electrophilic character with strong nucleophilic TME 15, and the reactions of tetrazines 14f–h with strong electrophilic TCE 18, have been studied within MEDT by using DFT calculations at the MPW95/6-311G(d,p) computational level. These reactions are domino processes comprising three consecutive steps: (i) formation of a MC at an early step of the process; (ii) an ADA reaction from this MC yielding a bicyclic compound; and finally, (iii) an extrusion of molecular nitrogen from this bicyclic compound affording the final dihydropyridazine. The ADA reaction is the RDS of these domino processes, with the bicyclic compounds not being observable.

The strong electrophilic character of tetrazines together with the strong nucleophilic character of TME 15 account for the high polar character of these ADA reactions, and consequently, for the reduction of the activation enthalpies in a polar process. A very good correlation between the GEDT at the polar TSs and the reduction of the activation enthalpy associated with these ADA reactions is observed, indicating that the electronic stabilisation of the polar TSs plays an important role in the accelerations found in these polar REDF ADA reactions. However, the FEDF ADA reactions of tetrazines 14f–h with the strong electrophilic TCE 18 present a very high activation enthalpy, in spite of the polar character of the reactions. These unfavourable activation enthalpies indicate that the tetrazine core has no tendency to participate as a nucleophile in FEDF ADA reactions. The extrusion of molecular nitrogen is less dependent on the EW substitution on the tetrazine core.

Analysis of the TS geometries associated with these ADA reactions indicates that they are associated to a synchronous C–C bond formation process, the distances between the interacting carbons being in the short range of 2.10–2.34 Å. An ELF topological analysis of the bonding changes along the ADA reaction between dinitro tetrazine 14a and TME 15 indicates that the activation energy is mainly associated to the continuous depopulations of the bonding regions of the double bonds present in the reagents.

## Conflicts of interest

There are no conflicts to declare.

## Supplementary Material

RA-010-D0RA01548B-s001
