# Supplementary material for: A molecular electron density theory study of the participation of tetrazines in aza-Diels–Alder reactions
Source: RSC Adv. 2020 Apr 21;10(26):15394–405. doi: 10.1039/d0ra01548b (PMC9052947; doi:10.1039/d0ra01548b)
Supplement: RA-010-D0RA01548B-s001 [file RA-010-D0RA01548B-s001.pdf]

Electronic Supplementary Material (ESI) for RSC Advances.
This journal is © The Royal Society of Chemistry 2020

Electronic Supporting Information

A Molecular Electron Density Theory Study of the Participation of Tetrazines in Aza-Diels-Alder Reactions

Luis R. Domingo,[1*] Mar Ríos-Gutiérrez,[1,2] and Patricia Pérez[3*]

[1] Department of Organic Chemistry, University of Valencia, Dr. Moliner 50, 46100 Burjassot, Valencia, Spain.
[2] Department of Chemistry and Chemical Biology, McMaster University, 1280 Main Street West, Hamilton, Ontario L8S 4L8, Canada
[3] Universidad Andres Bello, Facultad de Ciencias Exactas, Departamento de Ciencias Químicas, Computational and Theoretical Chemistry Group, Av. República 498, 8370146 Santiago, Chile.
E-mail: domingo@utopia.uv.es; p.perez@unab.cl

Index

S2 Figure with the geometries of **MCa** and **MCh**.

S3 Figure with the attractor positions of the ELF valence basins of the relevant IRC structures of the ADA reaction between dinitro tetrazine **14a** and TME **15**.

S4 Figure with the attractor positions of the ELF valence basins of the **TS1a-h**.

S5 Table with MPW95/6-311G(d,p) Global CDFT reactivity indices for the studied reagents.

S6 Tables with the MPW95/6-311G(d,p) electronic energies, enthalpies, entropies and Gibbs free energies for the stationary points involved in the domino reactions of the series tetrazine derivatives **14a-h** with TME **15**.

S8 Table with the MPW95/6-311G(d,p) electronic energies, enthalpies, entropies and Gibbs free energies for the stationary points involved in the domino reactions of the series tetrazine derivatives **14f-h** with TCE **18**.

S9 Table with the MPWB1K/6-311G(d,p) unique imaginary frequency of **TS1a-k** and **TS2a-k**.

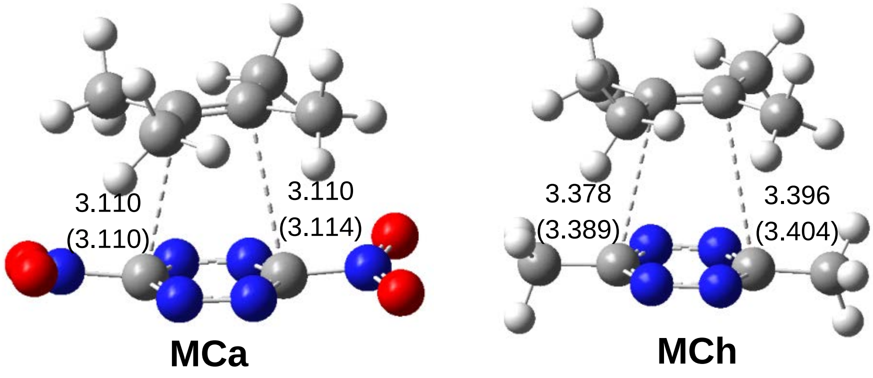

Figure S1. MPMWB95/6-311(d,p) geometries of the MCs **MCa** and **MCh**. The distances are given in Angstroms. Values in DCM are given in parentheses.

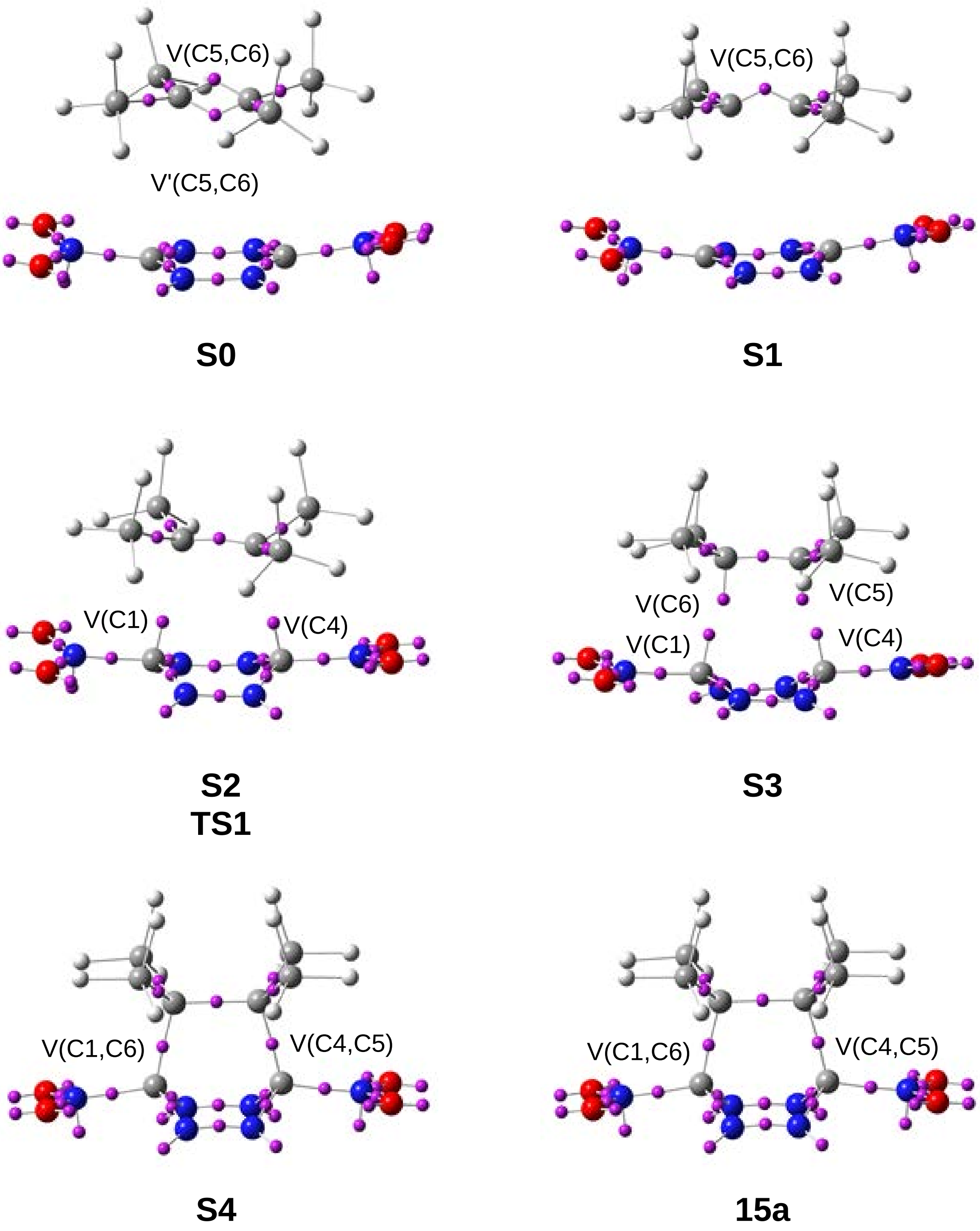

Figure S2. Attractor positions of the ELF valence basins of the IRC structures, **S0 – S4**, defining the five phases characterizing the molecular mechanism of the polar ADA reaction between dinitro tetrazine **14a** and TME **15**, **TS1a** and **15a** are also included.

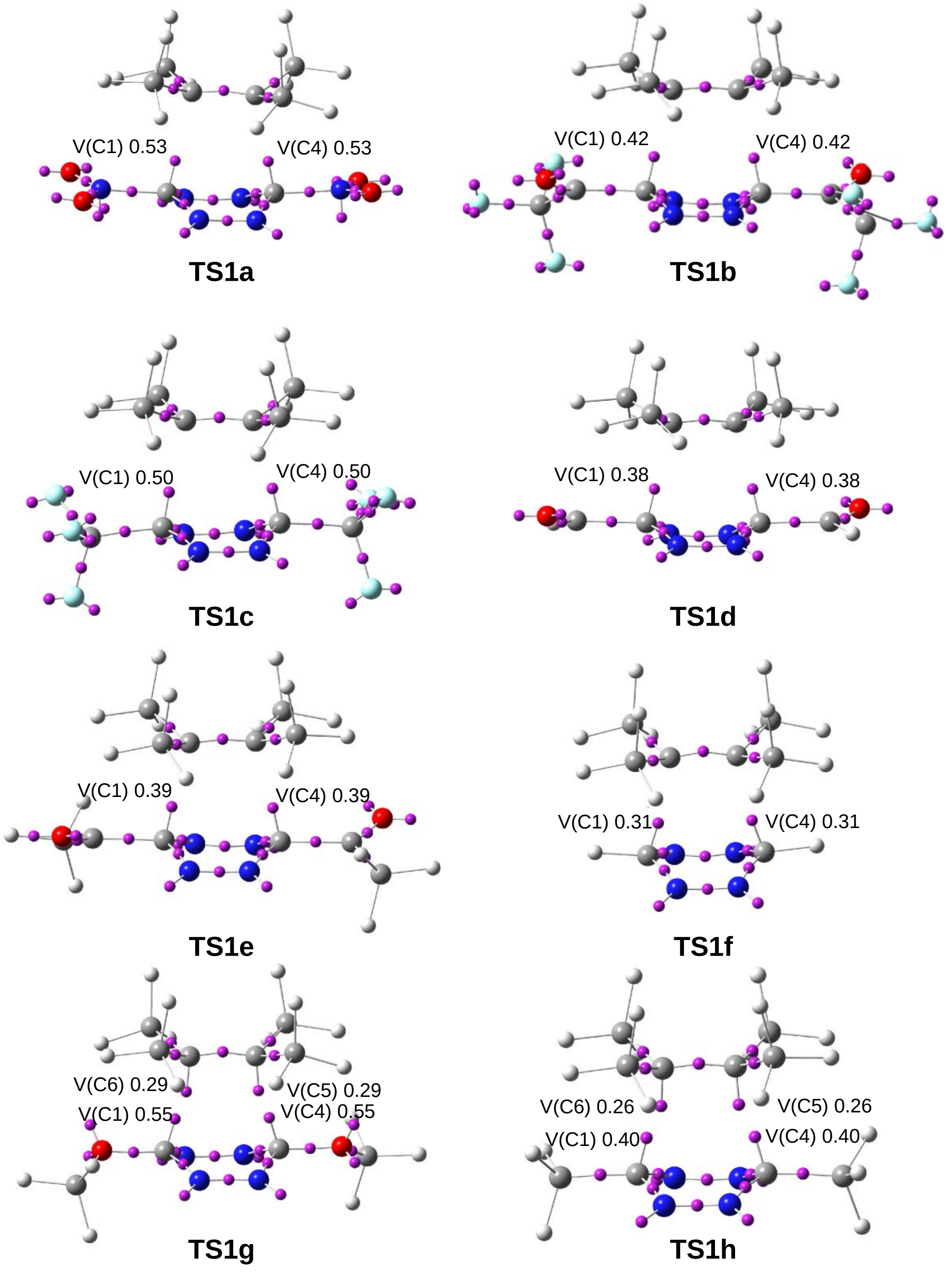

Figure S3. Attractor positions of the ELF valence basins of the **TS1a-h**. The electron populations, in average number of electrons, is given in e.

Table S1. MPW95/6-311G(d,p) electronic chemical potential μ, chemical hardness η, global electrophilicity ω, and global nucleophilicity *N*, in eV, for the studied reagents.

	R	μ	η	ω	*N*
TCE **18**		-7.35	6.09	4.44	0.00
**14a**	NO_2	-6.92	6.01	3.98	0.47
**14b**	$COCF_3$	-6.50	5.56	3.80	1.11
**14c**	CF_3	-6.25	6.03	3.24	1.13
**14d**	CHO	-5.98	5.74	3.12	1.54
**14e**	COMe	-5.55	5.85	2.63	1.92
**14f**	H	-5.17	6.03	2.22	2.21
**14g**	OMe	-4.94	5.91	2.07	2.50
**14h**	Me	-4.75	5.90	1.91	2.70
Ethylene **2**		-3.67	9.90	0.68	1.78
TME **15**		-2.64	8.85	0.40	3.33

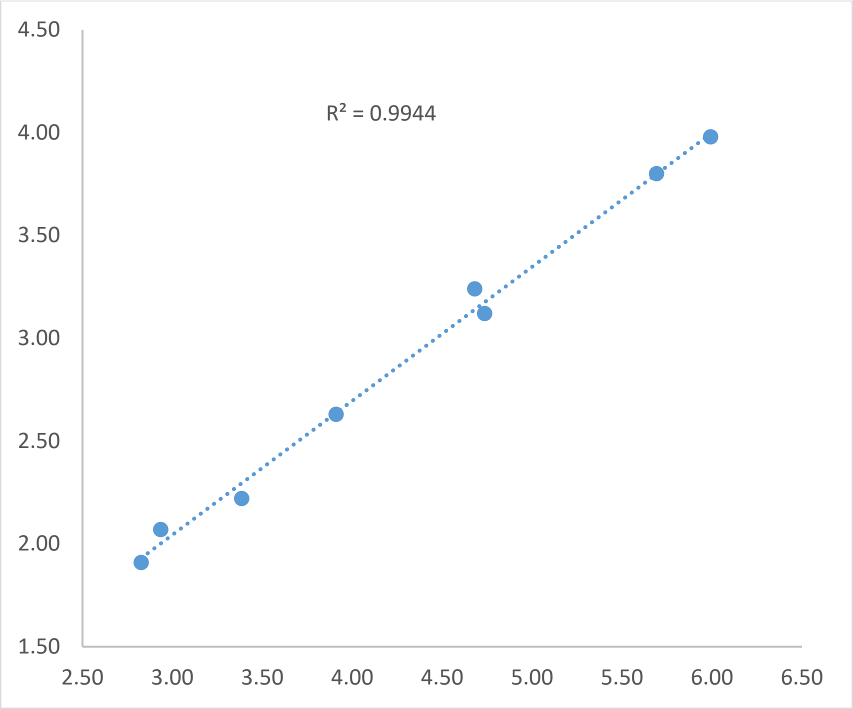

Figure S4. Lineal correlation between the B3LYP/6-31G(d) and the MPW95/6-311G(d,p) global electrophilicity ω indices of tetrazines **14a-h**.

Table S2. MPW95/6-311G(d,p) electronic energies (E, in a.u.), enthalpies (H, in a.u.), entropies (S, in cal/mol.K) and Gibbs free energies (G, in a.u.), computed at 25 ºC and 1 atm in DCM, for the stationary points involved in the domino reactions of the series tetrazine derivatives **14a-d** with TME **15**.

	E	H	S	G
**15**	-235.762490	-235.587197	83.6	-235.626919
**N2**	-109.503466	-109.494303	45.7	-109.516020
**14a**	-705.149383	-705.080595	97.9	-705.127111
**Mca**	-940.922268	-940.674856	139.2	-940.741013
**TS1a**	-940.912397	-940.666115	125.2	-940.725597
**16a**	-940.970454	-940.720548	120.3	-940.777724
**TS2a**	-940.943065	-940.695819	123.5	-940.754490
**17a**	-831.529971	-831.292073	121.6	-831.349837
**14b**	-1196.907292	-1196.808467	127.2	-1196.868915
**MCb**	-1432.679571	-1432.401999	172.1	-1432.483772
**TS1b**	-1432.660915	-1432.384802	155.7	-1432.458802
**16b**	-1432.710327	-1432.430657	147.2	-1432.500576
**TS2b**	-1432.687818	-1432.410889	152.4	-1432.483282
**17b**	-1323.276623	-1323.008965	150.5	-1323.080485
**14c**	-970.314012	-970.239771	106.1	-970.290182
**MCc**	-1206.086454	-1205.832324	149.2	-1205.903192
**TS1c**	-1206.062581	-1205.809544	132.8	-1205.872633
**16c**	-1206.123165	-1205.867072	130.6	-1205.929146
**TS2c**	-1206.097969	-1205.844381	130.6	-1205.906414
**17c**	-1096.683435	-1096.439244	128.8	-1096.500435
**14d**	-522.842940	-522.761816	90.3	-522.804719
**MCd**	-758.613679	-758.355212	133.1	-758.418447
**TS1d**	-758.591740	-758.333398	120.8	-758.390792
**16d**	-758.640010	-758.378397	114.6	-758.432846
**TS2d**	-758.620460	-758.361296	119.3	-758.417958
**17d**	-649.199426	-648.949699	117.1	-649.005329

Table S3. MPW95/6-311G(d,p) electronic energies (E, in a.u.), enthalpies (H, in a.u.), entropies (S, in cal/mol.K) and Gibbs free energies (G, in a.u.), computed at 25 ºC and 1 atm in DCM, for the stationary points involved in the domino reactions of the series tetrazine derivatives **14e-h** with TME **15**.

	E	H	S	G
**14e**	-601.469316	-601.329645	103.5	-601.378816
**Mce**	-837.241281	-836.923300	150.4	-836.994750
**TS1e**	-837.216513	-836.898310	133.3	-836.961668
**16e**	-837.265269	-836.943881	127.4	-837.004412
**TS2e**	-837.243974	-836.925040	132.5	-836.987995
**17e**	-727.828883	-727.519393	130.1	-727.581208
**14f**	-296.250369	-296.191733	68.0	-296.224042
**MCf**	-532.019808	-531.782661	118.8	-531.839099
**TS1f**	-531.992498	-531.756747	103.4	-531.805878
**16f**	-532.050019	-531.810586	98.4	-531.857330
**TS2f**	-532.023820	-531.787310	99.5	-531.834573
**17f**	-422.601918	-422.375955	94.6	-422.420890
**14g**	-525.274277	-525.143621	93.1	-525.187838
**MCg**	-761.044139	-760.734621	137.7	-760.800025
**TS1g**	-761.004197	-760.696710	125.1	-760.756165
**16g**	-761.059871	-760.750670	119.2	-760.807294
**TS2g**	-761.045961	-760.737557	121.1	-760.795116
**17g**	-651.638842	-651.339490	117.7	-651.395400
**14h**	-374.869781	-374.753117	83.8	-374.792915
**MCh**	-610.638501	-610.344321	125.7	-610.404055
**TS1h**	-610.602603	-610.307359	114.6	-610.361792
**16h**	-610.657750	-610.359038	109.8	-610.411208
**TS2h**	-610.633376	-610.337438	113.1	-610.391199
**17h**	-501.216475	-500.930536	106.8	-500.981266

Table S4. MPW95/6-311G(d,p) electronic energies (E, in a.u.), enthalpies (H, in a.u.), entropies (S, in cal/mol.K) and Gibbs free energies (G, in a.u.), computed at 25 ºC and 1 atm in DCM, for the stationary points involved in the domino reactions of the series tetrazine derivatives **14f-h** with TCE **18**.

	E	H	S	G
**18**	-447.412441	-447.353755	93.7	-447.398282
**14f**	-296.250369	-296.191734	68.0	-296.224044
**MCi**	-743.671571	-743.552230	129.4	-743.613714
**TS1i**	-743.609098	-743.492206	112.5	-743.545636
**19i**	-743.660889	-743.540928	110.0	-743.593187
**TS2i**	-743.639276	-743.522318	111.7	-743.575377
**20i**	-634.221429	-634.114355	107.4	-634.165384
**14g**	-525.274277	-525.143619	93.1	-525.187836
**MCj**	-972.696831	-972.505410	153.6	-972.578394
**TS1j**	-972.631587	-972.442820	135.7	-972.507281
**19j**	-972.667580	-972.477027	136.6	-972.541921
**TS2j**	-972.657040	-972.468551	136.0	-972.533160
**20j**	-863.255869	-863.075764	131.7	-863.138355
**14h**	-374.869781	-374.753117	83.8	-374.792915
**MCk**	-822.292731	-822.115362	140.6	-822.182181
**TS1k**	-822.234947	-822.059083	126.8	-822.119334
**19k**	-822.280144	-822.102589	118.0	-822.158641
**TS2k**	-822.259275	-822.083558	127.5	-822.144158
**20k**	-712.845780	-712.679087	120.5	-712.736340

Table S5. MPWB1K/6-311G(d,p) unique imaginary frequency of the TSs **TS1a-k** and **TS2a-k** computed in DCM, in cm^{-1}.

**TS1a**	-286.2929
**TS1b**	-337.4865
**TS1c**	-350.5034
**TS1d**	-379.2771
**TS1e**	-389.2782
**TS1f**	-432.0035
**TS1g**	-474.5200
**TS1h**	-473.2847
**TS1i**	-432.0035
**TS1j**	-568.1225
**TS1k**	-548.4563
**TS2a**	-582.5346
**TS2b**	-583.5926
**TS2c**	-576.9181
**TS2d**	-579.7784
**TS2e**	-578.0920
**TS2f**	-616.9326
**TS2g**	-597.8970
**TS2h**	-600.4108
**TS2i**	-619.0927
**TS2j**	-578.7566
**TS2k**	-601.7070
